# Illness Experience and Quality of Life in Sjögren Syndrome Patients

**DOI:** 10.3390/ijerph191710969

**Published:** 2022-09-02

**Authors:** Gonzalo Rojas-Alcayaga, Andrea Herrera, Iris Espinoza, Matías Rios-Erazo, Jacqueline Aguilar, Loreto Leiva, Nailah Shakhtur, Pamela Wurmann, Rinie Geenen

**Affiliations:** 1 Behavioral Science Area, Institute for Research in Dental Science, Faculty of Dentistry, Universidad de Chile, Santiago 8380544, Chile; 2 Dental and Maxillofacial Service, Clinical Hospital, Universidad de Chile, Santiago 8380456, Chile; 3 Department of Oral Medicine and Pathology, Faculty of Dentistry, Universidad de Chile, Santiago 8380544, Chile; 4 Department of Psychology, Faculty of Social Sciences, Universidad de Chile, Santiago 7800284, Chile; 5 National Association of Sjögren Patients of Chile, Santiago 8320214, Chile; 6 Reumathology Section, Medicine Department, Clinical Hospital, Universidad de Chile, Santiago 8380456, Chile; 7 Department of Psychology, Utrecht University, 3584 CS Utrecht, The Netherlands

**Keywords:** Sjögren’s syndrome, quality of life, women

## Abstract

Sjögren’s syndrome (SS) is a disease with autoimmune features that affects mainly women and compromises the health-related quality of Life (HRQoL); it is important to evaluate illness experience for a better understanding of the life situation of the patient. The aim of the study was to summarize the individual life experiences and determine the impact of HRQoL and oral health-related quality of life (OHRQoL) and their correlation with health self-assessment in women with SS. The life experiences evaluation employed a concept mapping design to structure qualitative content obtained from semi-structured interviews. Hierarchical cluster analysis was used to analyze the patient’s experiences. EQ-5D-5L and OHIP-14Sp were used. The correlation between appreciation of the general health status and OHIP-14 was evaluated. The experience classification by patients were analyzed and a dendrogram was obtained, identifying 10 clusters of disease experiences of SS, being limitations, pain and difficulties, coping and attitudes towards treatment the most common. Pain/discomfort in EQ-5D-5L and physical pain and psychological discomfort in OHIP-14 were the most affected dimensions in the patients. The results support the theoretical perspective that the experience of illness is relevant to describing the main difficulties of patients with SS and how it affects their quality of life.

## 1. Introduction

Sjögren’s syndrome (SS) is a disease with autoimmune features characterized by mononuclear cell infiltration of exocrine glands, notably the lacrimal and salivary glands; these lymphoid infiltrations lead to dryness of the eyes (keratoconjunctivitis sicca), dryness of the mouth (xerostomia), and frequently, dryness of other surfaces connected to exocrine glands [1]. Epidemiological studies have revealed discrepant prevalences, ranging from 0.01% to more than 3% of the general population; considering only primary Sjögren’s syndrome (pSS), the estimated prevalence was 0.25% (95% CI 0.15–0.43) or 1 person in 400 [2]. A systematic review and meta-analysis showed a female/male ratio in prevalence data of 10.72 (95% CI 7.35 to 15.62) and the overall age of pSS patients of 56.16 years (95% CI 52.54 to 59.78) [3].

In recent years, the heterogeneity of clinical presentations among newly diagnosed adults with SS has been better appreciated. Approximately 80% of the overall patient group presents some form of the sicca syndrome [4]. Musculoskeletal manifestations such as myalgia, arthralgia and morning stiffness are present in as many as 90% of the patients, while clinically evident arthritis is found in up to 17% [5]. The prevalence of mood disorders is higher, which is associated with symptom burden and disability; 33.8% of SS patients had anxiety and 36.9% had depression [6]. SS patients scored high in neuroticism and anxiety and low in sociability [7]. Health-related Quality of Life is defined as “how well a person functions in their life and his or her perceived wellbeing in physical, mental, and social domains of health” [8]. According to this definition, patients with SS have lower Health-related Quality of Life (HRQoL) than the general or healthy population, specifically physical and mental functioning components of HRQoL are reduced [9].

These findings highlight the importance of evaluating and knowing how patients with SS live their illness, make sense of it and respond to the adversities of their disease. In a previous study on experiences of Sjögren’s disease in Chilean women, we concluded that both biomedical and psychosocial aspects are of vital importance for the health of patients with SS, identifying ten clusters arranged in six main categories: ‘Symptoms’, ‘Social environment’, ‘Emotion management’, ‘Information’, ‘Coping strategy’, and ‘Health staff relationship’ [10]. Although illness experiences are best understood with qualitative analysis of SS, questionnaires could provide information for better communication with patients and to learn how they cope with the disease. To our knowledge, research that includes both qualitative study about illness experience and quantitative analysis of quality of life in women with Sjögren’s syndrome, has not been previously developed in Chile.

The first aim of the present study was to structure and summarize the individual life experiences of Chilean women with SS in an integrated model using a concept mapping method. Secondarily, we aimed at determining the degree of agreement with the experiences of illness among patients with SS. The third aim was to determine the impact on health-related quality of life (HRQoL) and oral health-related quality of life (OHRQoL) and their correlation with the current health self-assessment in women with SS.

## 2. Materials and Methods

### 2.1. Ethics

The research was approved by the Ethics Committee of the Faculty of Dentistry of the University of Chile on 14 December 2016 (ethical approval code: 2016/43). Everybody signed an informed consent form before the research began.

### 2.2. Sample

Participants were recruited through a Facebook message by the national association of Sjögren patients of Chile (SjögrenChile), the Facebook group “Síndrome de Sjögren Chile” and through the treating physician at the University of Chile Clinical Hospital. The inclusion criteria were women between 18 and 70 years old with a medically confirmed diagnosis of SS. The sample consisted of 31 women between 29 and 68 years old, all with a medically confirmed diagnosis of SS by a rheumatologist and based on American College of Rheumatology/European League Against Rheumatism criteria [11], without distinguishing between primary or secondary SS. Exclusion criteria were pregnancy, other untreated chronic conditions, mental disorders and patients in the acute phase of SS.

The women were selected progressively, in order of arrival. An appointment was made with each participant, individually, to perform the card sorting task and request responses to two questionnaires, one about General Quality of Life named EuroQoL Spanish version (EQ-5D-5L) [12] and the second about Oral Health-related Quality of Life named Oral Health Impact Profile -14 Spanish version (OHIP-14Sp) [13].

### 2.3. Procedure

#### 2.3.1. Life Experiences of Chilean Women with SS

This study employed a concept mapping design to structure qualitative content obtained from semi-structured interviews performed by two psychologists (A.H and G.R), which examined experiences about glandular effects of SS, pain, and fatigue in fifteen (*n* = 15) women with SS, medically confirmed by a rheumatologist.

The qualitative content was converted into 70 cards with statements involving experiences of living with SS.

Besides, participants indicated their level of agreement (level of agreement task) related to their experiences on a 4-point Likert rating (agree, slightly agree, slightly disagree, disagree). Then, the participants categorized the cards into piles using similarity of content and gave each pile a label. To better understand the concept mapping technique and the procedure used in our study, consult previous research [10].

#### 2.3.2. Quality of Life

Participating women completed two questionnaires: EQ-5D-5L and OHIP-14Sp, and a visual analog scale about general health.

#### 2.3.3. General Quality of Life

EQ-5D-5L studies quality of life globally and has two parts. The first part has a descriptive system that includes 5 dimensions: mobility, self-care, habitual activities, pain/discomfort, and anxiety/depression and each dimension has 5 levels: no problems (score 0), minor problems (score 1), moderate problems (score 2), serious problems (score 3) and extreme problems (score 4). In the second part a visual analog scale (EQ-VAS) where different health states are scored on a scale with values from 0 to 100; 0 is considered “The worst health you can imagine” and 100 “The best health you can imagine” [14].

#### 2.3.4. Oral Health-Related Quality of Life

The OHIP-14Sp instrument, in its version validated in Chile [13], is a questionnaire with 14 questions, which assesses the negative impact of dental problems on quality of life related to oral health, covering 7 dimensions: functional limitation, physical pain, psychological discomfort, physical disability, psychological disability, social disability and handicap. The response levels in each question range from “never” to “always”, with a Likert scale of 5 options [15]. OHIP-14Sp can range between 0 and 56 points.

### 2.4. Statistical Analyses

Descriptive statistics were obtained to describe the sociodemographic variables (age, diagnosis, symptom duration, marital status and education level). For objective 1, hierarchical cluster analysis (Ward’s method, squared Euclidean distances) was used to analyze the experiences that were individually sorted by the participants. In cluster analysis, the cells of the input matrix of experiences comprised the number of times that two experiences were not sorted in the same pile. The number of clusters was set guided by the dendrogram and agglomeration schedule produced by the statistical software showing which experiences were being combined at each stage of the hierarchical clustering process. The main criterion to decide on the number of clusters was that they should reflect distinct components of experiences.

To analyze the level of agreement (objective 2), a non-parametric statistical test for one sample (Wilcoxon Signed Rank Test) was used to compare the response of the participants with the median (2.5) response possibility. Statistical significance of an item indicates that there was agreement among patients reflecting a common or uncommon experience. Based on the number of significant items in each cluster, the agreement percentage of each one of them was calculated. The median was derived to describe the agreement of the participants with the items.

Regarding objective 3, the frequency of responses was calculated for each item of each dimension of the EQ-5D-5L questionnaire. Likewise, the mean and deviation in the appreciation of the general health status identified with the visual scale (EQ-VAS) were obtained. For OHIP-14Sp results, an average score per dimension was calculated. The correlation between appreciation of the general health status identified with the visual scale (EQ-VAS) and the five dimensions of the EQ-5D-5L was evaluated, as well as the correlation between appreciation of the general health status and the seven dimensions of the OHIP-14, using Spearman’s correlation coefficient.

The IBM Statistical Software Package for the Social Sciences (SPSS) version 22 for Windows was used for all analyses. A *p*-value less than 0.05 was considered to indicate statistical significance.

## 3. Results

The median age of the participants in the semi-structured interviews was 55 years, with a minimum age of 29 years and a maximum of 68 years. The time elapsed since the diagnosis of the disease was between a minimum of 2 months and a maximum of 120 months, with a median of 12 months. Seven women only had a diagnosis of SS and 8 also reported having one or more other diseases (four rheumatoid arthritis, two fibromyalgia, two hypothyroidism, two hypertension, one lupus erythematosus, one diabetes).

The sociodemographic characteristics of the participants in the main part of the study are shown in Table 1. The women suffered from different symptoms and glandular or systemic signs of the disease. The more commonly self-reported symptoms by the patients were ocular and mouth dryness with 74.2% each. More than half of participants (51,6%) referred to muscular and joint pain, as well as fatigue 29%.

### 3.1. Objective 1: Concept Analysis

**Figure 1:**
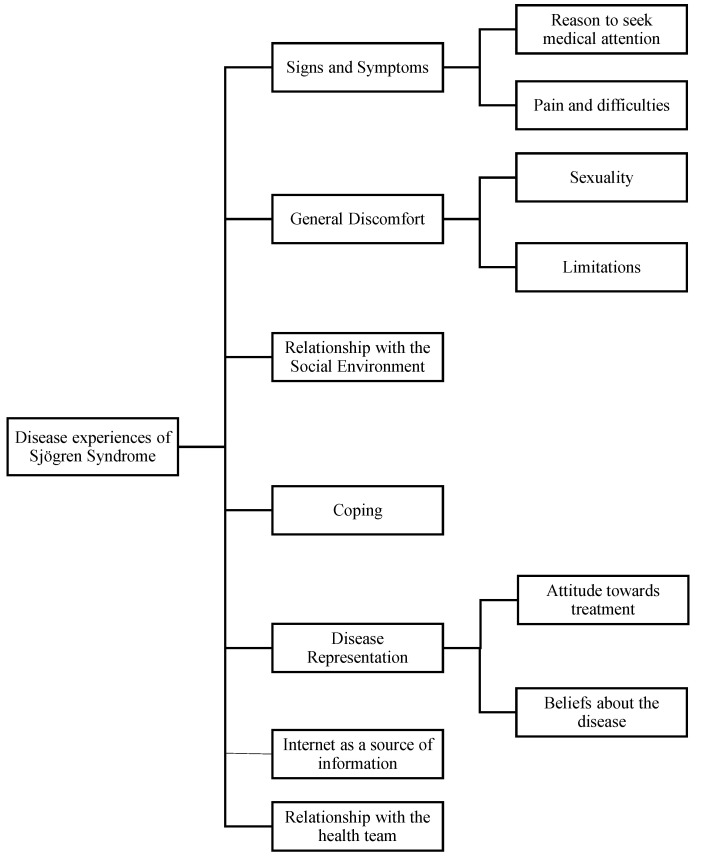
Schematic overview of the outcome of hierarchical cluster analysis grouping the 70 experiences of having SS.

The results of the classification of 70 experience cards by patients with Sjögren’s Syndrome were analyzed with hierarchical clustering and a dendrogram of the grouped experiences was obtained. The team-based consensus analysis determined that the number of clusters was set to 10 (Figure 1). Decreasing the number of clusters from 10 to 7 allowed for a solution combining three pairs of clusters in overarching categories.

The items included in the clusters are shown in Appendix A Table A1. The clusters ‘Reason to seek medical attention’ and ‘pain and difficulties’ were not combined although both clusters contain items about “signs and symptoms “of SS. The reasons for consulting a doctor (the first signs and symptoms of Sjögren’s Syndrome) are not necessarily found in patients who have established disease. For example, patients can be compensated by pharmacological treatment.

The patient’s experiences with Sjögren include the category ‘General discomfort’, and it was observed that there were clearly discernible experiences associated with ‘sexuality’ and ‘limitations’ and therefore it was determined that both groups were separated.

Also, the pairs of clusters ‘Attitude towards treatment’ and ‘Beliefs about the disease’ could be combined in overarching categories; however, it was decided that these separate clusters would be maintained due to their point to different topics. Finally, the experiences of having SS comprised on the highest-order level seven domains, three of which included two lower-order clusters each.

### 3.2. Objective 2: Level of Agreement with Experiences

Patients indicated their level of agreement with the 70 experiences associated with the illness. The score distributions are arranged according to their belonging to one of the higher-order dimensions (Table A1 Appendix A Affirmations of illness experiences). A higher level of common experiences was observed in the participants (62.9%, 44 experiences of illness). Clusters that describe the more common experiences among patients (higher than 70% of agreement) are “limitations” with 100%, “pain and difficulties” and “coping” with 89%, and “attitudes towards treatment” with 71%. The experiences with more agreement of those clusters are: *There are days when I wake up very well and suddenly I feel bad* (limitations), *even if I feel bad, I encourage myself and try to be independent* (coping), *I wish there were more treatment alternatives or that it was more comprehensive* and *The treatments are expensive* (attitudes towards treatment), and *I always need to eat sweets, gum or drink a lot of water* (pain and difficulties).

There were four clusters without a marked trend, that is, without significant agreements or disagreements; these were “internet as source of information” (50% of agreement) with only two significant agreement experience: *I don’t believe everything that I find on the internet* and *I have been learning about the disease through the internet*. Two of the experiences with more agreement of the cluster “relationships with the social environment” (50% agreement) were *Sometimes I look good on the outside, so people don’t understand that I’m sick*, and *People that don’t suffer this disease don’t understand it*. Cluster “beliefs about disease” (57% of agreement) had one significant disagreement experience *When I was diagnosed, I didn’t give it much importance* and one of the experiences with more agreement was *I had never heard of this disease before*. Finally, “Relationships with the health team” reached 46% of agreement with three significant disagreements experiences *The doctor told me that I don’t have to look for more information; I have not looked for more information, I only know what the doctor told me*, and *I didn’t dare to ask the doctor more about it.*

On the other hand, clusters “reason to seek medical attention” and “sexuality” are less common experiences with 0% and 33% of agreement each. In “Sexuality”, only the experience *Sometimes I don’t have the energy to have sex* was significant.

### 3.3. Objective 3: Quality of Life

Finally, the impact that SS generated on health quality of life (EQ-5D-5L and EQ-VAS) and oral health-related quality of life (OHIP 14sp) were determined, and the correlation between appreciation of the general health status with EQ-VAS and the score in the OHIP-14Sp questionnaire was measured.

Regarding EQ-5D-5L, the dimension “Self-care” stands out, in which 71% of the participants selected the option “I have no problem”, in addition to reporting low levels of “Anxiety/depression”. On the other hand, in the dimension “Pain/Discomfort”, when grouping the responses from mild to extreme pain, 87.1% presented some degree of pain (Table 2).

The EQ-VAS visual numerical scale gave an average of 67.32, with a standard deviation of 20.75, where the minimum chosen was a value of 10 and the maximum was 100 (the best health you can imagine).

The relationship between the EQ-VAS and the five dimensions of the EQ-5D-5L suggests that respondents’ EQ-5D-5L profiles show an indirect association in the expected direction (better current status in patients with no problem); however, only in three dimensions of EQ-5D-5L (mobility, usual activities and pain/discomfort) the correlation with EQ-VAS was statistically significant (*p* < 0.05) (Table 3).

In relation to OHIP-14sp, the sample obtained an average of 20.13 points in its total score, with a standard deviation of 13.08 and a minimum and maximum of 2 and 52 points respectively.

In relation to the classification of the dimensions, the averages of the scores yielded higher scores in the dimension “Physical pain” with 4.45 and in “Psychological discomfort” with 3.54 and “Psychological disability” 3.22 (Table 4).

In addition, the association between the current health status assessment and the 7 dimensions of the OHIP-14Sp questionnaire was evaluated. Regarding this analysis, a statistically significant and indirect correlation, in the expected direction, between EQ-VAS and two dimensions of OHIP-14Sp was observed: “Functional limitation” and “Psychological disability” (Table 5).

## 4. Discussion

Sjögren’s syndrome is a little-known disease by health professionals and the community in general. Additionally, there is limited research on the experience of illness and the quality of life of people who suffer from it; this research contributes to reducing this knowledge gap. Clusters that describe the more common experiences among patients are pain and difficulties, limitations, coping and attitudes towards treatment. The less common experiences were reasons to seek medical attention, sexuality and relationship with the health team. The dimension of the general quality of life in which a greater proportion of people is affected is “pain/discomfort”, and the least affected is “self-care”. The dimension of quality of life in oral health that shows the greatest negative impact is “physical pain”.

The main strength of this study is to approach an investigation with a vision that is as complete and general as possible, encompassing the objective with the subjective as a whole. Most of the research on SS has focused on the description of the syndrome, its treatment and its relationship with other diseases. A smaller percentage has focused on the effects on quality of life, and very few have delved into the experiences of suffering from this disease, or the significance that women attribute to the different symptoms.

Among the limitations of the study is the size of the sample, which does not allow the generalization of the results. The fact that the participants present other associated rheumatological diseases make it difficult or impossible to attribute the experiences of illness only to SS, since these other diseases may be affecting their perception of the disease and altering their quality of life; however, when these diseases occur together, they can be considered part of the same phenomenon; this study included only female participants, which on the one hand limits the global understanding of the disease but gives a gender perspective to the study, highlighting how SS affects the role of women; it should be taken into consideration that this disease affects women in a greater proportion, female/male ratio reported was 10.72 [3].

### 4.1. Experiences of Disease and Level of Agreement with Experiences

In a recent study about SS experiences in Chilean women, ten clusters were identified, arranged in six main categories: ‘Symptoms’ (clusters: ‘Mucosal dryness’ and ‘Related symptoms’), ‘Social environment’, ‘Emotion management’ (clusters: ‘Fears’ and ‘Sadness’), ‘Information’ (clusters: ‘Uncertainty’ and ‘Lack of knowledge’), ‘Coping strategy’ (clusters: ‘Resilience’ and ‘Self-care’), and ‘Health staff relationship’ [10]; these clusters are similar to those of our study, but two new clusters emerged, the Internet as a source of information and sexuality.

Searching information on the internet is a very important topic nowadays. Nevertheless, this cluster was not a common experience, reflecting that the Internet has a different impact on patients. Internet health information can provide a sense of empowerment, purpose, control, and patient satisfaction [16]. Internet health information allows patients control over their rate of learning, reducing information overload [17]. Other positive effects of Internet health information are enhanced patient confidence in dealing with physicians, better health choices and decision-making, improved understanding of health conditions, and improved communication with physicians [18,19].

Although sexuality emerges as a new cluster, it is not consolidated as a common experience. Since the experiences point only to the issue of pain in sexual relationships and do not address other important aspects, the in-depth interviews, from where the experiences emerged could be debatable; this is because they were not able to create the climate of trust, necessary to delve into the topic of sexuality, going beyond the physical limitations of vaginal dryness.

In the previous study in Chile, the cluster related to social environment was not a common experience between participants [10]. The experiences with a significant agreement mentioned the understanding and comprehension about the disease by the social environment. Examples of experiences are: The social environment neither knows nor understand the disease, and People that do not suffer this disease, do not understand it in our study.

The most common experiences into the cluster “relationships with the health team” were disagreement experiences, probably because the two experiences point to search for information and the vast majority of the participants do not agree to be limited in their search regarding the disease by the health team. In relation to this cluster and “Relationships with the health team”, we can say that both are related because Internet health information has an impact on the patient-physician relationship. Broom [16] states that the patient’s concern regarding health information on the internet is about the physician’s disapproval. Patients worry that this disapproval can lead to hostility from the physician, irritation, and lower quality of care resulting in patient anxiety, confusion, and frustration [16]. On the other hand, physicians worry that the use of the Internet may lead to patient confusion and unrealistic expectations [20].

Laugensen et al. [21] found that the patient’s perception about physician quality related to competence/knowledgeability, communication capabilities and empathy impacts directly on patient-physician concordance (agreement regarding the medical problem and its treatment) and information asymmetry (patient’s perception that the physician has a greater quantity and/or quality of information compared to themselves); furthermore, the physician quality impacts indirectly on compliance with the physician’s instructions. Additionally, they found that better Internet health information quality can lead to enhanced compliance.

### 4.2. General Measures of Health-Related Quality of Life

Regarding the general quality of life in patients with SS Sjogren, it has been determined that the physical dimensions are not routinely evaluated by the caregivers and it impacts on the psychological/emotional and social domains, due to the lack of understanding of the disease on the part of the family [22]. In accordance with these results, in our study we observed the emergence of clusters “Relationship with the health team” and “Relationship with the social environment”.

Various physical symptoms of SS, such as fatigue, dry eyes and vaginal dryness have been associated with an impact on quality of life. Fatigue is negatively associated with quality of life in patients with primary SS [23]; however, one study found an improvement in vitality, which could be explained by the effectiveness of the coping strategies implemented by the patients [24]; these conclusions highlight the importance of the “coping” cluster, obtained in this research, being one of the clusters with a higher percentage of agreement in relation to the experiences. In relation to the symptom of dry eye characteristic of SS, the more its severity increases, the more the quality of life is affected, both in relation to their perception of health, social and physical functioning, as well as limitations in the emotional sphere [25]. With regard to sexual dysfunction, particularly in patients with vaginal dryness, different studies report a low quality of life [26,27]. Although in this study the cluster “sexuality” did not present agreement in relation to common experiences of dryness or pain in sexual intercourse, its emergence as a cluster implies its relevance and it should be considered in future investigations.

In relation to psychology, there is a history that shows that high levels of anxiety and depression in patients with primary SS are associated with low levels of quality of life [28,29]. For example, a high level of psychological distress is presented in patients with primary SS, compared to patients with another autoimmune disease such as Systemic Lupus Erythematosus, and to a control group without these diseases [30].

Regarding the quality of life measured through generic scales, such as the SF-36 or the EuroQol-5, studies in different countries, such as Spain, the Netherlands, the United States, and Turkey, among others, reveal a low quality of life in patients with primary SS, compared to healthy controls or to the general population [31]. Lendrem et al. [32] determined that the pain and anxiety/depression dimensions in the EQ-5D were the dimensions most affected in the quality of life of patients with SS; it should be noted that in our study the “pain and difficulties” cluster was one of the clusters with a higher level of common experiences among the participants.

Qualitative studies in relation to the quality of life in patients with SS are scarce. Ngo et al. [9] found that quality of life is negatively affected by the long time elapsed until an accurate diagnosis is obtained, by the low quality of care from the health professionals, and the lack of ability of the patients themselves to positively face this chronic disease.

### 4.3. Specific Measures of Oral Health-Related Quality of Life

The OHRQoL in patients with SS appears to be frequently impaired. In the present study, the average of the total summations of the scores was 20.13 points, a figure comparable with the averages found in other studies that evaluate OHRQoL in patients with SS that ranging between 11.3 points [33] and 23.7 points [34]. Furthermore, these values are considerably higher than those of the general population, ranging from 5.7 to 8.5 points. So, the signs and symptoms of this disease would have a negative impact on the OHRQoL in comparison with healthy individuals [34].

The OHRQoL in patients with rheumatic disease, not only SS, appears to be frequently impaired. Systematic literature research including rheumatoid arthritis (RA, seven studies), systemic sclerosis (SSc, five), Sjögren syndrome (SS, eight), Behcet disease (BD, four), systemic lupus erythematosus (SLE, one) and ankylosing spondylitis (AS, one), found that the majority of studies (14/15) reported worse score in OHRQoL in patients with rheumatic disease compared to healthy individuals. In particular, patients with SS (salivary flow and composition) or BD (oral ulcers) showed a relation between OHRQoL and disease-specific oral manifestations. In this review, most studies that investigated subscales of OHRQoL (5/6) found the subscale physical disability to be predominantly affected in patients with rheumatic diseases and about half of the studies reported impaired psychosocial aspects [35]. In the analysis by dimensions, our study also highlights the dimensions related with psychological aspects had a high negative impact on patients with SS. Psychological discomfort and psychological disability dimensions of OHIP-14 were the second and third dimension most affected in our study with SS patients, respectively, only after the dimension “Physical pain”. Regarding pain between the dimensions of OHQoL, it has been described that hyposialie causes a lower buffering capacity against acids, so that by decreasing the pH of the mouth the possibilities of dental sensitivity would increase [36].

The main oral problems studied that could affect SS patients are xerostomia, hyposialie and oral lesions. In a study with 61 patients with SS, 92% percent reported xerostomia, 61% suffered hyposialie and 35% presented oral mucosa lesions [37]. Dry mouth/salivary flow has been associated with a negative impact measured with OHIP-14 [34]. Disaggregated by OHIP-14 dimensions, a negative correlation has been observed between physical pain, physical disability dimensions with stimulated and differential salivary flows; and a negative correlation between unstimulated salivary flow with physical pain [38]; however, it is important to mention that the OHIP14-Sp questionnaire is a generic tool that does not incorporate the specific oral conditions affected by the syndrome, so the oral problems present in the patients may not be sufficiently represented.

Findings of our study with OHIP-14 and previous published research underscored the importance of oral manifestations of SS patients and their impact on maintaining quality of life.

### 4.4. Meaning of the Study for Clinicians and Policy Makers

The visibility of the syndrome in health professionals would allow, among other benefits: greater recognition for a correct referral or early diagnosis, the handling of more information to guide the patient and their family, greater empathy and understanding by the health team with these patients and the possibility of proposing more comprehensive treatments.

Currently there is no comprehensive treatment for this disease, which addresses psychological, gynecological, ophthalmological, dental and rheumatic aspects. Patients must see different specialists separately. Patients report that professionals sometimes do not have a clear vision of what the syndrome is about, due to its low prevalence; this article provides a comprehensive vision of the disease that gives policy makers a background on the requirement of a multidisciplinary team for the basic treatment of this disease.

### 4.5. Unresolved Questions and Future Research

In relation to the questionnaires used in this research to evaluate quality of life, they have different limitations, one of them being their generic nature: they measure dimensions such as mobility, personal care, daily activities, pain/discomfort and presence of anxiety and depression, so that it can be used both in relatively healthy individuals (general population) and in groups of patients with different diseases [14]. The use of generic instruments of quality of life in relation to health makes it possible to compare the results with other diseases and the general population, and thus makes the SS visible; however, there are dimensions of the experience that are not evaluated in this type of questionnaire (and that showed relevance in our study) such as the issue of sexuality, the relationship with the family, understanding and social support regarding the disease; this is particularly relevant in the clinical setting, so that the treating physician can determine the best possible treatment scheme based on the information provided by the specific quality of life questionnaires, such as the PSS-QoL, which was created especially for patients with primary Sjogren’s syndrome [22].

It is important to indicate that there are other variables that may influence the results obtained (such as marital status or level of education); however, these were not part of the objectives of the study, so we consider it relevant to include in future, more quantitative research on this theme.

## 5. Conclusions

The results support the theoretical perspective that the experience of illness in patients with SS affects quality of life.

Although the experience of disease is unique in each person, we detected that in a high percentage of patients there was a concordance, so these experiences should be considered when describing the disease and proposing possible palliative treatments.

The quality of life in relation to general health is affected, especially with a negative impact on mobility, usual activities and pain/discomfort.

An association was observed between current health status and quality of life in relation to oral life among the patients, in the dimensions of functional limitation and psychological disability.

Physical pain, psychological discomfort and psychological disability dimensions of OHIP-14 were the most affected dimensions in our study with SS patients.

## Figures and Tables

**Table 1:** Characteristics of the sample.

Characteristic (*n* = 31)	Mean (SD), Range or Number and Percentage (%)
Age in years	43.5 (13.38), range = 21–68
Age of onset of the first symptoms	34.8 (12.06), range = 17–59
Age of diagnosis	38.5 (11.44), range = 20–60
Symptom duration before Diagnosis	3.2 (3.91), range = 0–11
Marital status	
Married or cohabiting	13 (41.9)
Divorced	6 (19.4)
Widowed	2 (6.5)
Single	10 (32.3)
Highest level of completed education	
Incomplete secondary education	4 (12.9)
Complete secondary education	7 (22.6)
Incomplete university education	2 (6.5)
Complete university education	14 (45.2)
Postgraduate	4 (12.9)
Self-report of symptoms	
Eye dryness	23 (74.2)
Oral dryness	23 (74.2)
Muscle/Joint pain	16 (51.6)
Fatigue	9 (29.0)
Parotiditis	6 (19.4)
Vaginal dryness	5 (16.1)
Dry skin	4 (12.9)
Respiratory problems	3 (9.8)
Eye problems	3 (9.8)
Dry nose	3 (9.8)
Dental problems	2 (6.5)
Dysphagia	1 (3.2)

**Table 2:** Frequency and percentage of results of the EQ-5D-5L in each dimension.

Dimension	Item	*n* (%)
Mobility	I have no problems walking about	15 (48.4)
	I have slight problems walking about	9 (29.0)
	I have moderate problems walking about	6 (19.4)
	I have severe problems walking about	1 (3.2)
	I am unable to walk about	0
Self-care	I have no problems washing or dressing myself	22 (71.0)
	I have slight problems washing or dressing myself	6 (19.4)
	I have moderate problems washing or dressing myself	3 (9.7)
	I have severe problems washing or dressing myself	0
	I am unable to wash or dress myself	0
Usual activities	I have no problems doing my usual activities	12 (38.7)
	I have slight problems doing my usual activities	9 (29.0)
	I have moderate problems doing my usual activities	6 (19.4)
	I have severe problems doing my usual activities	2 (6.5)
	I am unable to do my usual activities	2 (6.5)
Pain/discomfort	I have no pain or discomfort	4 (12.9)
	I have slight pain or discomfort	8 (25.8)
	I have moderate pain or discomfort	15 (48.4)
	I have severe pain or discomfort	3 (9.7)
	I have extreme pain or discomfort	1 (3.2)
Anxiety/depression	I am not anxious or depressed	14 (45.2)
	I am slightly anxious or depressed	7 (22.6)
	I am moderately anxious or depressed	6 (19.4)
	I am severely anxious or depressed	3 (9.7)
	I am extremely anxious or depressed	1 (3.2)

**Table 3:** Correlation between EQ-VAS with the five dimensions of the EQ-5D-5L.

EQ-5D-5L Dimensions	Correlation Coefficient	*p* Value
Mobility	−0.507	0.004 ^1^
Self-care	−0.311	0.088
Usual activities	−0.691	<0.05 ^1^
Pain/discomfort	−0.589	<0.05 ^1^
Anxiety/depression	−0.347	0.056

^1^ Pearson correlation or Spearman (*p* ≤ 0.05).

**Table 4:** Averages and standard deviation of OHP-14sp score in each dimension.

OHIP-14sp Dimensions	Average	Standard Deviation	95% CI
Functional limitation	3.12	1.65	2.54–3.73
Physical Pain	4.45	2.42	3.56–5.34
Psychological discomfort	3.55	2.79	2.52–4.57
Physical disability	2.04	2.47	1.19–3.00
Psychological disability	3.22	2.22	2.41–4.03
Social disability	2.19	2.56	1.25–3.13
Handicap	1.48	2.42	0.60–2.37

**Table 5:** Correlation between health status measure with ES-VAS and OHIP-14Sp score.

OHIP-14Sp	Correlation Coefficient	*p* Value
Score sum	−0.343	0.059
Dimensions		
Functional limitation	−0.361	0.046 ^1^
Physical pain	−0.171	0.357
Psychological discomfort	−0.224	0.225
Physical disability	−0.19	0.305
Psychological disability	−0.415	0.02 ^1^
Social disability	−0.27	0.142
Handicap	−0.194	0.296

^1^ Pearson correlation (*p* ≤ 0.05).

## Data Availability

Not applicable.

## Appendix A

**Table A1:** Affirmations of illness experiences.

My Experience with Sjögren’s Syndrome Is That	Agree	Somewhat Agree	Somewhat Disagree	Disagree	*p*
(1) A doctor sent me to another professional.	51.6 (16)	16.1 (5)	6.5 (2)	25.8 (8)	0.08
(2) I had never heard of this disease before.	83.9 (26)	0 (0)	3.2 (1)	12.9 (4)	<0.001 ^1^
(3) I try not to think about the disease or talk about it.	35.5 (11)	38.7 (12)	19.4 (6)	6.5 (2)	0.004 ^1^
(4) I have not had support with my illness.	16.1 (5)	25.8 (8)	12.9 (4)	45.2 (14)	0.081
(5) The doctor told me that I don’t have to look for more information.	9.7 (3)	19.4 (6)	6.5 (2)	64.5 (20)	0.001 ^1^
(6) I don’t understand some information from the internet.	22.6 (7)	19.4 (6)	19.4 (6)	38.7 (12)	0.264
(7) Sometimes I want to stop (or have stopped) the medication due to adverse reactions.	35.5 (11)	16.1 (5)	6.5 (2)	41.9 (13)	0.779
(8) I don’t know how I will feel tomorrow.	74.2 (23)	9.7 (3)	0 (0)	16.1 (5)	0.001 ^1^
(9) My doctor doesn’t inform me enough about my illness.	22.6 (7)	32.3 (10)	9.7 (3)	35.5 (11)	0.678
(10) Some days I can’t open my eyes and the sun bothers them.	54.8 (17)	29 (9)	6.5 (2)	9.7 (3)	<0.001 ^1^
(11) The pain in my eyes motivated myself to ask to the doctor.	29 (9)	12.9 (4)	6.5 (2)	51.6 (16)	0.187
(12) I didn’t dare to ask the doctor more about it.	19.4 (6)	6.5 (2)	25.8 (8)	48.4 (15)	0.023 ^1^
(13) People that don’t suffer this disease, don’t understand it.	80.6 (25)	9.7 (3)	3.2 (1)	6.5 (2)	<0.001 ^1^
(14) Even if I feel bad, I encourage myself and try to be independent.	87.1 (27)	9.7 (3)	3.2 (1)	0 (0)	<0.001 ^1^
(15) When I have a lot of pain I can’t do housework and my daily routine.	58.1 (18)	19.4 (6)	3.2 (1)	19.4 (6)	0.008 ^1^
(16) It has a hereditary component.	22.6 (7)	25.8 (8)	16.1 (5)	35.5 (11)	0.485
(17) It bothers me to be constantly taking medicine, using drops, etc.	67.6 (21)	22.6 (7)	6.5 (2)	3.2 (1)	<0.001 ^1^
(18) I think that my illness will never end, that I will always be sick.	74.2 (23)	16.1 (5)	0 (0)	9.7 (3)	<0.001 ^1^
(19) I don’t say it and I don’t explain it to anyone because it is difficult to understand.	29 (9)	32.3 (10)	12.9 (4)	25.8 (8)	0.492
(20) This permanent disease generates other diseases.	58.1 (18)	19.4 (6)	16.1 (5)	6.5 (2)	<0.001 ^1^
(21) I don’t research on websites because there is information that I don’t like or scares me.	25.8 (8)	22.6 (7)	12.9 (4)	38.7 (12)	0.489
(22) I plan several things to do, but for fatigue, I only finish one.	48.4 (15)	25.8 (8)	3.2 (1)	22.6 (7)	0.041 ^1^
(23) I have isolated myself socially.	22.6 (7)	25.8 (8)	6.5 (2)	45.2 (14)	0.244
(24) I live tired, living tired is very uncomfortable.	51.6 (16)	22.6 (7)	9.7 (3)	16.1 (5)	0.010 ^1^
(25) I have lowered my efficiency in my daily activities.	45.2 (14)	41.9 (13)	9.7 (3)	3.2 (1)	<0.05 ^1^
(26) When I was diagnosed, I felt relieved because I already knew what I had.	41.9 (13)	3.2 (1)	16.1 (5)	38.7 (12)	0.958
(27) I feel joint pain and fatigue.	71 (22)	16.1 (5)	3.2 (1)	9.7 (3)	<0.001 ^1^
(28) It is very difficult to ask for an appointment with the doctor or specialist.	54.8 (17)	16.1 (5)	3.2 (1)	25.8 (8)	0.052
(29) With and without medication I feel the same, or worse.	25.8 (8)	9.7 (3)	22.6 (7)	41.9 (13)	0.194
(30) I can’t eat dry foods.	54.8 (17)	9.7 (3)	22.6 (7)	12.9 (4)	0.01 ^1^
(31) I got used to living with pain.	38.7 (12)	29 (9)	12.9 (4)	19.4 (6)	0.085
(32) The treatments are expensive.	83.9 (26)	9.7 (3)	3.2 (1)	3.2 (1)	<0.001 ^1^
(33) There are doctors and dentists who don’t know the disease.	54.8 (17)	19.4 (6)	6.5 (2)	19.4 (6)	0.014 ^1^
(34) I want, but i can’t cry.	48.4 (15)	6.5 (2)	16.1 (5)	29 (9)	0.275
(35) My doctor has always been very willing and responsive.	54.8 (17)	16.1 (5)	16.1 (5)	12.9 (4)	0.005 ^1^
(36) I feel pain in my legs.	67.7 (21)	16.1 (5)	9.7 (3)	6.5 (2)	<0.001 ^1^
(37) I always need to eat sweets, gum or drink a lot of water.	80.6 (25)	16.1 (5)	0 (0)	3.2 (1)	<0.001 ^1^
(38) The system is hostile; they punish you for being sick.	29 (9)	25.8 (8)	9.7 (3)	35.5 (11)	0.895
(39) Sometimes I don’t have the energy to have sex.	61.3 (19)	25.8 (8)	3.2 (1)	9.7 (3)	<0.001 ^1^
(40) Sometimes I look good on the outside, so people don’t understand that I’m sick.	83.9 (26)	3.2 (1)	6.5 (2)	6.5 (2)	<0.001 ^1^
(41) Sometimes doctors don’t believe you.	41.9 (13)	19.4 (6)	9.7 (3)	29 (9)	0.319
(42) This disease is activated on the emotional side.	67.7 (21)	19.4 (6)	6.5 (2)	6.5 (2)	<0.001 ^1^
(43) When I feel bad, I try not to let them realize it so as not to worry them.	54.8 (17)	16.1 (5)	12.9 (4)	16.1 (5)	0.010 ^1^
(44) My family is always aware of my illness.	54.8 (17)	9.7 (3)	19.4 (6)	16.1 (5)	0.018 ^1^
(45) Those who work with patients are unkind and don’t put themselves in our shoes.	22.6 (7)	32.3 (10)	22.6 (7)	22.6 (7)	0.785
(46) I have not looked for more information, I only know what the doctor told me.	12.9 (4)	9.7 (3)	9.7 (3)	67.7 (21)	0.001 ^1^
(47) I don’t believe everything that I find on the internet.	45.2 (14)	32.3 (10)	6.5 (2)	16.1 (5)	0.011 ^1^
(48) I have been learning about the disease through the internet.	32.3 (10)	45.2 (14)	9.7 (3)	12.9 (4)	0.013 ^1^
(49) There are days when I wake up very well and suddenly, I feel bad.	71 (22)	19.4 (6)	3.2 (1)	6.5 (2)	<0.001 ^1^
(50) I thought that dry mouth was normal, and not a symptom of this disease.	61.3 (19)	16.1 (5)	3.2 (1)	19.4 (6)	0.006 ^1^
(51) As long as I take care of myself, everything will be fine, because it depends on me.	45.2 (14)	29 (9)	22.6 (7)	3.2 (1)	0.001 ^1^
(52) My teeth have been badly affected.	61.3 (19)	12.9 (4)	6.5 (2)	19.4 (6)	0.008 ^1^
(53) My partner has been understanding about the consequences of the disease in sexual relations.	54.8 (17)	12.9 (4)	6.5 (2)	25.8 (8)	0.062
(54) I avoid having sex because of my vaginal dryness.	35.5 (11)	29 (9)	9.7 (3)	25.8 (8)	0.286
(55) I wish there were more treatment alternatives or that it was more comprehensive.	93.5 (29)	6.5 (2)	0 (0)	0 (0)	<0.001 ^1^
(56) When I found out about the diagnosis, I was sad.	61.3 (19)	19.4 (6)	6.5 (2)	12.9 (4)	0.001 ^1^
(57) I have continued my normal life; I have not deprived myself due to the illness.	41.9 (13)	41.9 (13)	6.5 (2)	9.7 (3)	0.001 ^1^
(58) It bothers me to think that this is not going to end.	61.3 (19)	22.6 (7)	3.2 (1)	12.9 (4)	0.001 ^1^
(59) I don’t ask for help, nor do I tell how I feel, so as not to bother.	38.7 (12)	25.8 (8)	12.9 (4)	22.6 (7)	0.167
(60) You must face people and say things clearly.	41.9 (13)	22.6 (7)	25.8 (8)	9.7 (3)	0.022 ^1^
(61) I feel that doctors don’t have time to attend.	29 (9)	22.6 (7)	19.4 (6)	29 (9)	0.943
(62) I was motivated to see the doctor because of my dry eyes.	32.3 (10)	19.4 (6)	3.2 (1)	45.2 (14)	0.546
(63) I feel burning and dry eyes.	74.2 (23)	16.1 (5)	6.5 (2)	3.2 (1)	<0.001 ^1^
(64) They tell you about the symptoms you might have, but it is different when you really live it.	74.2 (23)	16.1 (5)	6.5 (2)	3.2 (1)	<0.001 ^1^
(65) There is no effective treatment that will assure you that it will work.	67.7 (21)	16.1 (5)	9.7 (3)	6.5 (2)	<0.001 ^1^
(66) When I was diagnosed, I didn’t give it more importance.	19.4 (6)	16.1 (5)	9.7 (3)	54.8 (17)	0.029 ^1^
(67) It hurts me to have sex.	35.5 (11)	19.4 (6)	22.6 (7)	22.6 (7)	0.401
(68) What motivated me to see the doctor was my pain in the extremities and joints.	45.2 (14)	6.5 (2)	9.7 (3)	38.7 (12)	0.719
(69) I have to do what the doctors say and take care of myself.	48.4 (15)	38.7 (12)	9.7 (3)	3.2 (1)	<0.001 ^1^
(70) Illness happens to you because it is your turn.	35.5 (11)	19.4 (6)	16.1 (5)	29 (9)	0.618

^1^ Wilcoxon Signed Rank Test respect 2.5 (*p* ≤ 0.05).
